# Deciphering sepsis: An observational bioinformatic analysis of gene expression in granulocytes from GEO dataset GSE123731

**DOI:** 10.1097/MD.0000000000040559

**Published:** 2024-11-15

**Authors:** Li Jin, Xiaowei He, Yuanyuan Wang, Feng Shao, Jun Qian, Mengxiao Jiang, Shengjie Zhang, Wenjie Liao

**Affiliations:** a Department of Emergency, Nantong Third People’s Hospital, Affiliated Nantong Hospital 3 of Nantong University, Nantong, Jiangsu, China; b Department of Emergency Medicine, The First Affiliated Hospital of Soochow University, Suzhou, Jiangsu, China; c Department of Emergency, Affiliated Rugao Hospital of Nantong University Xinglin College, Rugao People’s Hospital, Rugao, Jiangsu, China; d Department of Emergency, Lianyungang Second People’s Hospital Affiliated to Kangda College of Nanjing Medical University, Lianyungang, Jiangsu, China.

**Keywords:** bioinformatics, differential gene expression, neutrophils, sepsis

## Abstract

Sepsis triggers severe inflammatory responses leading to organ dysfunction and demands early diagnostic and therapeutic intervention. This study identifies differentially expressed genes (DEGs) in sepsis patients using the Gene Expression Omnibus database to find potential diagnostic and therapeutic markers. We analyzed the dataset GSE123731 via GEO2R to detect DEGs, constructed protein–protein interaction networks, and performed transcription factor analyses using Cytoscape. Gene Ontology and Kyoto Encyclopedia of Genes and Genomes pathway analyses were conducted using R and FunRich software. Key genes were validated by Quantitative Reverse Transcription Polymerase Chain and co-immunoprecipitation assays in granulocytes from sepsis patients. We identified 59 DEGs significantly involved in neutrophil degranulation and immune system activation. Cytokine signaling pathways were highlighted in Kyoto Encyclopedia of Genes and Genomes analysis. Co-immunoprecipitation assays confirmed interactions involving matrix metallopeptidase 8, matrix metallopeptidase 9, and arginase 1, supporting their roles as biomarkers. The identified DEGs and validated interactions reveal crucial molecular mechanisms in sepsis, offering new avenues for diagnostic and therapeutic strategies, potentially enhancing patient outcomes.

## 1. Introduction

Sepsis is a life-threatening condition triggered by an infection that leads to multi-organ failure.^[1]^ Clinically, it progresses through a spectrum that starts with Systemic Inflammatory Response Syndrome (SIRS) and can escalate to severe sepsis and septic shock, the latter associated with mortality rates of approximately 30% to 50%.^[2,3]^ Early differentiation of noninfectious SIRS from septic conditions early is critical for the timely administration of antibiotics, which can significantly improve patient outcomes.^[4]^ Despite this, the early detection of sepsis remains a significant clinical challenge.^[5]^ Traditional bacteriological testing is often supplemented by whole blood genomic sequencing, a cutting-edge method that offers a new way to differentiate between bacterial infections and sterile inflammations, thus enhancing our understanding of sepsis’ pathophysiology and aiding in the identification of reliable biomarkers.^[6,7]^

During the acute inflammatory phase of sepsis, there is a marked increase in the production of neutrophils, accompanied by a decrease in lymphocytes and monocytes.^[8]^ These neutrophils produce substantial quantities of reactive oxygen species and reactive nitrogen species, causing both reversible and irreversible damage to proteins, lipids, and DNA, which ultimately impairs cellular functions and contributes to organ damage.^[9]^ The genomic sequencing of these neutrophils has provided valuable insights into the pathogenesis of sepsis and has been instrumental in the identification of dependable biomarkers.

With the rapid advancement in gene-chip technology and high-throughput sequencing in recent years, researchers can conduct more detailed studies on the differential genes and their precise mechanisms in sepsis. However, focused research specifically on gene chips related to sepsis remains scarce. This study employs the public health database, Gene Expression Omnibus (GEO), to download the GSE123731 dataset, which includes samples from control, SIRS, and septic shock groups. Our objective is to identify differentially expressed genes, analyze these genes through Gene Ontology (GO) and Kyoto Encyclopedia of Genes and Genomes (KEGG) pathways, and examine the transcription factors involved. We then aim to validate target genes are validated using Quantitative Reverse Transcription Polymerase Chain Reaction, providing potential molecular markers for the early diagnosis and treatment of sepsis. This multifaceted approach not only broadens our understanding of sepsis at the molecular level but also contributes significantly to the development of novel diagnostic and therapeutic strategies.

## 2. Materials and methods

### 2.1. Dataset download and analysis

The dataset GSE123731, obtained from the GEO database, includes transcriptomic data from granulocytes of 11 healthy controls, 16 patients diagnosed with Systemic Inflammatory Response Syndrome (SIRS), and 15 patients experiencing septic shock. This comprehensive dataset encompasses a broad participant base reflecting typical clinical scenarios. This dataset utilizes the GPL21970 platform. Procedures for data normalization and the identification of differentially expressed genes (DEGs) were executed using GEO2R, with the criteria being |logFC| > 2 and an adjusted *P*-value < .05.

### 2.2. Protein–protein interaction network and core gene selection

The DEGs were subjected to protein–protein interaction (PPI) analysis using the STRING database (https://string-db.org). Subsequent interaction networks were visualized with Cytoscape software (version 3.5.1). Additionally, literature-based PPI networks for the DEGs were formulated using the GenCLiP 2.0 database (http://cismu.net/GenCLiP2/analysis.php). Co-immunoprecipitation (CoIP) assays: To validate the protein-protein interactions identified through bioinformatics analysis, CoIP assays were performed. Protein extracts from granulocytes isolated from sepsis patients were prepared using cell lysis buffer containing protease inhibitors. Antibodies specific to matrix metallopeptidase 8 (MMP8), matrix metallopeptidase 9 (MMP9), arginase 1 (ARG1), and C–X–C motif chemokine ligand 8 (CXCL8) were used to pull down the respective proteins. The immunoprecipitates were then analyzed by Western blot to detect the presence of target proteins and their interactors. This method allowed for the confirmation of direct physical interactions among the proteins, providing robust support for the bioinformatic predictions. Core genes within these networks were pinpointed using the MCODE (version 1.5.1) and cytoHubba (version 0.1) plugins in Cytoscape, with an MCODE score threshold set at >3.

### 2.3. GO, KEGG pathway, and transcription factor analysis

GO and KEGG pathway analyses were performed using R software (version 4.0.3) equipped with the clusterProfiler and enrichGO packages. Statistical significance was determined with a *P*-value < .05 and a *q*-value < 1. Identification of transcription factors associated with the DEGs was executed using the iRegulon plugin in Cytoscape (version 1.3), with a normalized enrichment score threshold set at >7.

### 2.4. Patient recruitment and ethical considerations

Patients for the study were recruited from the emergency ICU of Nantong Third People’s Hospital following the third International Consensus Definitions for Sepsis and Septic Shock. Exclusion criteria included pregnancy, recent cardiopulmonary resuscitation, corticosteroid therapy, end-stage renal disease, liver disease, and a history of organ transplantation. This subset included 8 patients from both the SIRS and septic shock groups, along with 8 healthy controls from a wellness center, distinct from the broader dataset initially described. This study was conducted in accordance with the ethical standards of the institutional and national research committee and with the 1964 Helsinki declaration and its later amendments or comparable ethical standards. The ethical approval for this study was granted by the Ethics Committee of Nantong Third People’s Hospital (Approval number: EL2022023). Informed consent was obtained from all individual participants included in the study.

This study involves 2 levels of participant data: the initial broad dataset from GEO for exploratory bioinformatic analysis and a specifically recruited subset for in-depth experimental validations. The selection of a smaller, focused group from the larger dataset was driven by the need to control for variables more effectively in our experimental assays and to ensure the reproducibility and precision of our findings. This approach allowed us to conduct detailed analyses of key interactions within a representative but smaller group of participants, enhancing the scientific rigor of our experimental conclusions.

### 2.5. Target gene validation

Within 24 hours of hospital admission, 10 mL of whole blood was drawn from each participant. CD15+ granulocytes were then isolated using CD15+ immunomagnetic beads (Miltenyi Biotec, Germany). RNA extraction was carried out using the Trizol method (Thermo Fisher Scientific), followed by conversion to cDNA synthesis using a kit from Novozymes (Nanjing, China). Quantitative PCR was performed using SYBR Green on an ABI7500 system (Applied Biosystems). The primers used were: MMP8 forward: ccctggtgccttgatgt, reverse: gtttgggtgtgcttggtc; MMP9 forward acgcagacatcgtcatcc, reverse ccagggaccacaactcg; and ARG1 forward ggaagtgaacccatccct, reverse gattaccctcccgagca, with beta-actin acting as an internal control. Data expression was quantified using the 2−ΔΔCt method, and statistical significance was determined by an independent *t* test (*P* < .05) using SPSS 20.0 software.

## 3. Results

### 3.1. Differential gene selection

**Figure 1.**
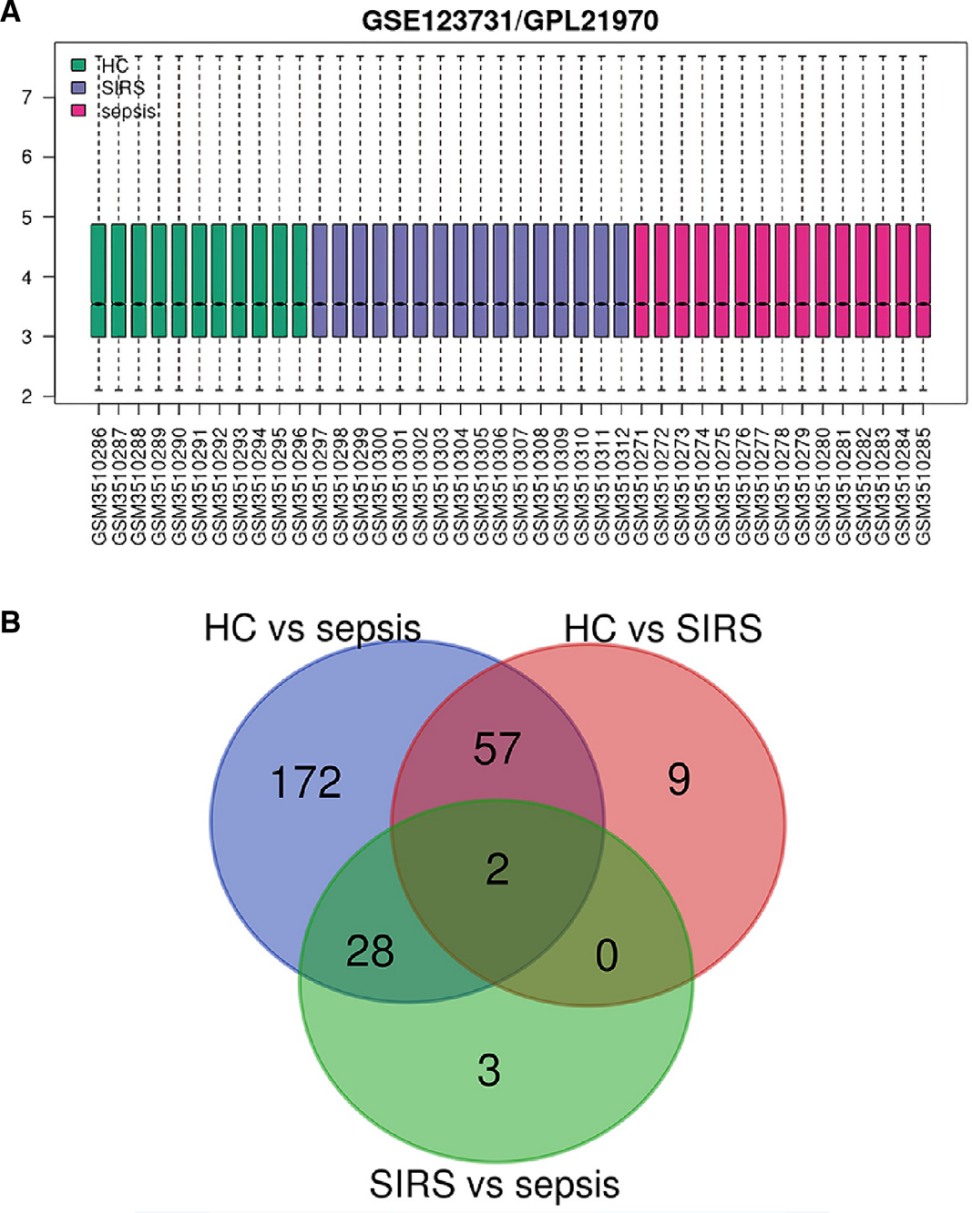
Gene expression profiling and DEG distribution in sepsis. (A) Box plot demonstrating the normalization of gene expression levels across 3 study groups in the GSE123731 dataset. Each box represents the interquartile range (IQR) of expression levels for healthy controls (HC), systemic inflammatory response syndrome (SIRS), and septic shock patients, with the median expression level indicated by the horizontal line within the box. (B) Venn diagram illustrating the overlap of differentially expressed genes (DEGs) between healthy controls (HC), SIRS, and septic shock groups. The numbers indicate the count of unique and shared DEGs between the compared groups, highlighting a core subset of 59 DEGs common to all groups.

Post-normalization analysis of the GSE123731 dataset revealed distinct gene expression profiles among the study groups (Fig. 1A). The septic shock group displayed 259 DEGs when compared to the control group, with 152 upregulated and 107 downregulated genes. Conversely, the SIRS group presented 68 DEGs relative to the control group, which included 40 upregulated and 28 downregulated genes. A subsequent comparison between the septic shock and SIRS groups yielded 33 DEGs, with 25 upregulated and 8 downregulated. Importantly, a core subset of 59 DEGs was consistently detected in the septic shock, SIRS, and control groups, consisting of 25 upregulated and 24 downregulated genes (Fig. 1B).

### 3.2. Protein–protein interaction network and core gene selection

**Figure 2.**
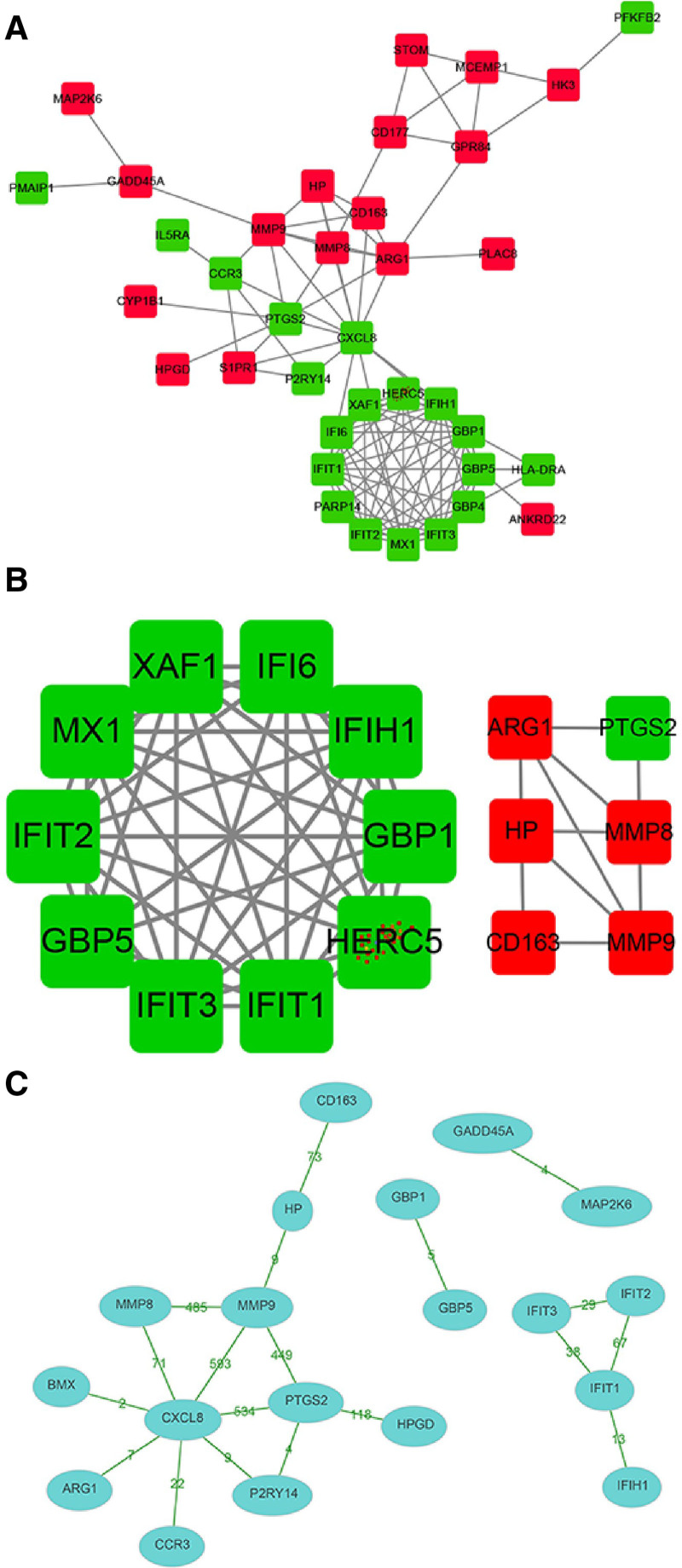
Analysis of protein–protein interactions and core gene networks in sepsis. (A) Protein–protein interaction (PPI) network of differentially expressed genes (DEGs) involved in sepsis. Nodes represent genes, with upregulated genes shown in red and downregulated genes in green. Edges indicate the interactions between proteins encoded by these genes, highlighting the interconnected nature of the inflammatory response in sepsis. (B) Core interaction network identifying hub genes within the PPI network. Nodes represent hub genes, with upregulated genes in red and downregulated genes in green, interconnected by thick lines indicating strong associations. This network was derived using the MCODE algorithm, showcasing the most interconnected and potentially influential genes in the network. (C) Co-citation network for DEGs highlighting the number of research articles supporting the interactions, with MMP9 shown to have extensive connections, particularly with MMP8 and PTGS2. Nodes are sized according to the number of citations, demonstrating the evidence strength behind reported interactions.

Among the DEGs, 37 were found to be involved in forming PPI networks, with 17 being upregulated and 20 downregulated (Fig. 2A). The use of MCODE for network analysis identified 16 hub genes, comprising 5 upregulated and 11 downregulated (Fig. 2B). Particular attention was given to 5 genes—MMP8, MMP9, ARG1, HP, and CD163—that were upregulated in both septic shock and SIRS conditions. MMP9, notably, exhibited extensive interactions, especially with MMP8 and PTGS2, as indicated by evidence from 485 cited articles (Fig. 2C).

**Figure 3.**
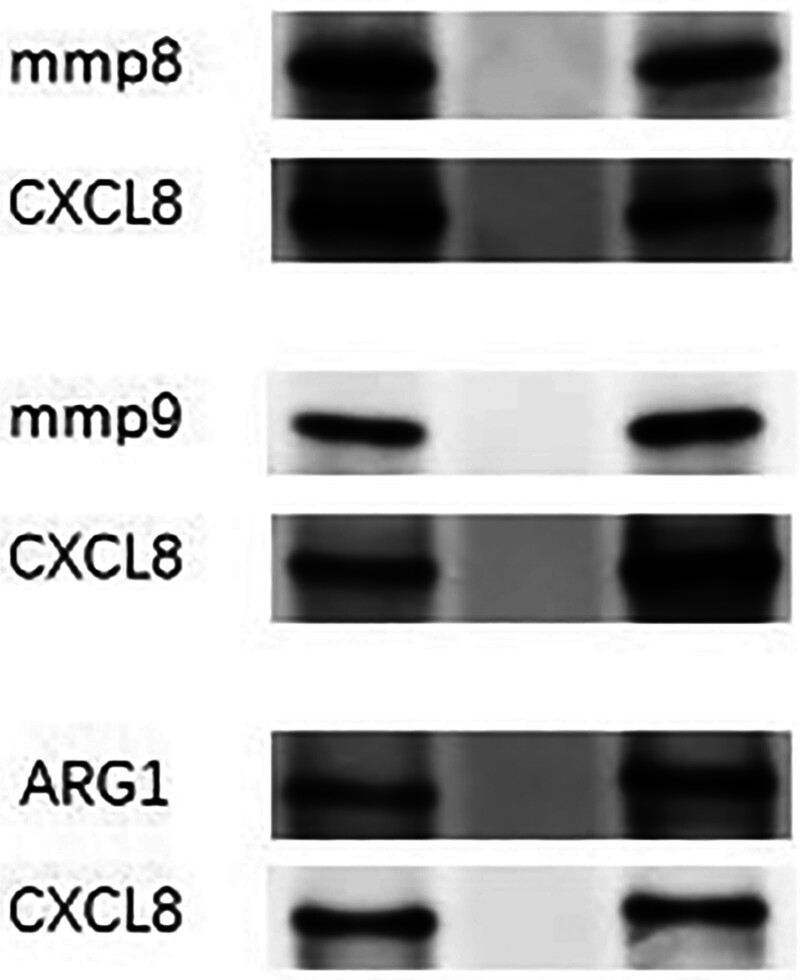
Co-immunoprecipitation assays confirming interactions between sepsis biomarkers. Panel A: MMP8 and CXCL8 interaction—Western blot showing the immunoprecipitation of MMP8 followed by the detection of CXCL8, confirming their direct interaction in the context of sepsis. Panel B: MMP9 and CXCL8 interaction—Western blot results demonstrating the co-immunoprecipitation of MMP9 with CXCL8, indicating a functional interaction that may influence inflammatory responses in sepsis. Panel C: ARG1 and CXCL8 interaction—shows the successful pull-down of ARG1 and subsequent detection of CXCL8, highlighting their interaction which could play a role in modulating arginine metabolism during septic conditions.

To further validate the protein–protein interactions identified in our bioinformatic analysis, CoIP assays were performed. These assays specifically aimed to confirm the interactions between key inflammatory mediators identified in our study. The CoIP results confirmed direct interactions between MMP8, MMP9, ARG1, and CXCL8, substantiating their roles in sepsis-related inflammatory pathways. These findings provide robust experimental support for our PPI network predictions and highlight potential targets for therapeutic intervention in sepsis (Fig. 3).

### 3.3. Target gene validation

**Figure 4.**
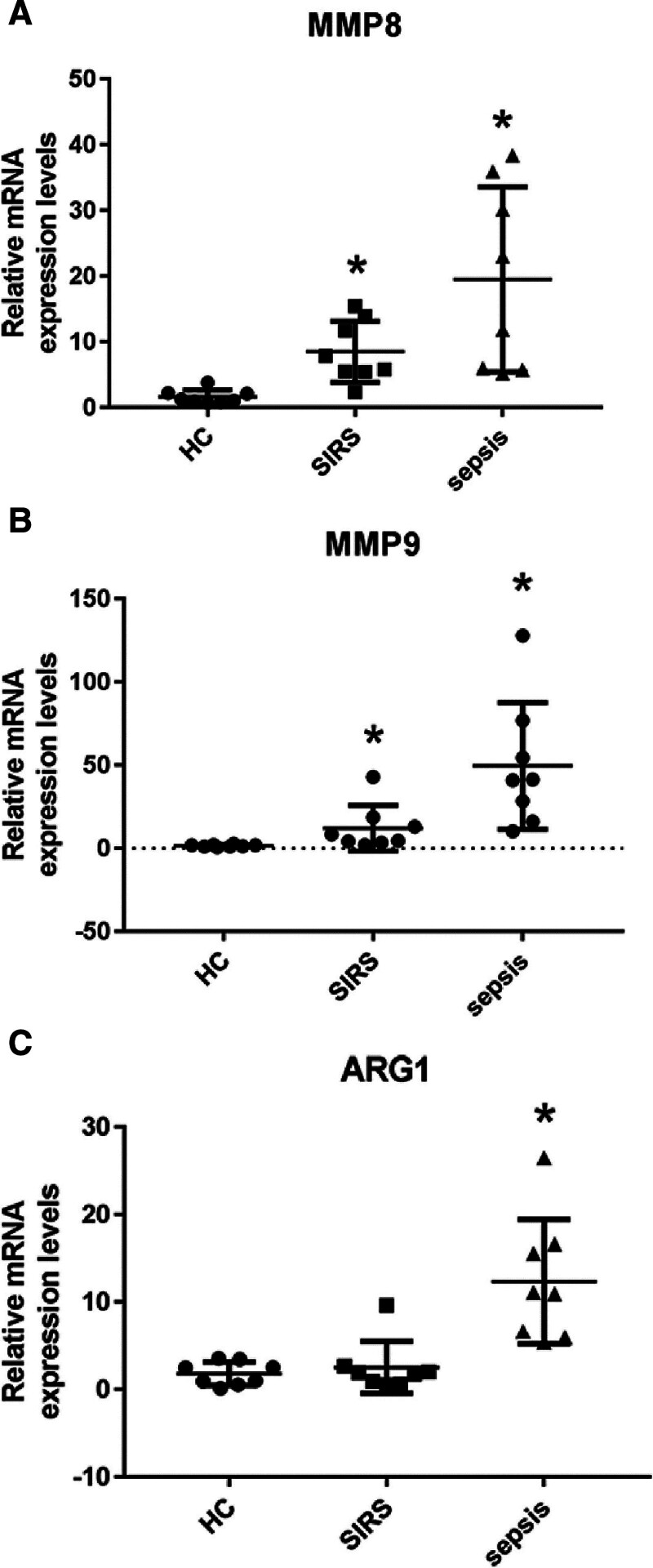
Gene expression validation in granulocytes across patient groups. (A) Scatter plot depicting the relative mRNA expression levels of MMP8 in granulocytes from healthy controls (HC), systemic inflammatory response syndrome (SIRS), and septic shock patients. Each point represents an individual sample, and the bars indicate the median with interquartile range. There is a significant upregulation of MMP8 expression in the SIRS and septic shock groups compared to HC, as denoted by the asterisk (*). (B) Scatter plot illustrating the relative mRNA expression levels of MMP9 in granulocytes across the same patient groups. Similar to MMP8, MMP9 expression is significantly elevated in the SIRS and septic shock groups in comparison to HC, indicated by the asterisk (*). (C) Scatter plot showing the relative mRNA expression levels of ARG1 in granulocytes from the 3 groups. ARG1 expression is significantly higher in septic shock patients compared to HC, with no significant difference between the SIRS group and HC, as represented by the asterisk (*) marking the septic shock group.

Quantitative Reverse Transcription Polymerase Chain Reaction validation was conducted for MMP8, MMP9, ARG1, HP, and CD163 across healthy controls, SIRS, and septic shock patient groups. The results verified significant upregulation of MMP8 and MMP9 in both the SIRS and septic shock groups compared to the controls (Fig. 4A and B). Moreover, ARG1 expression was significantly elevated in the septic shock group compared to controls, whereas no marked difference was observed in the SIRS group (Fig. 4C).

### 3.4. GO, KEGG pathway, and transcription factor analysis

**Figure 5.**
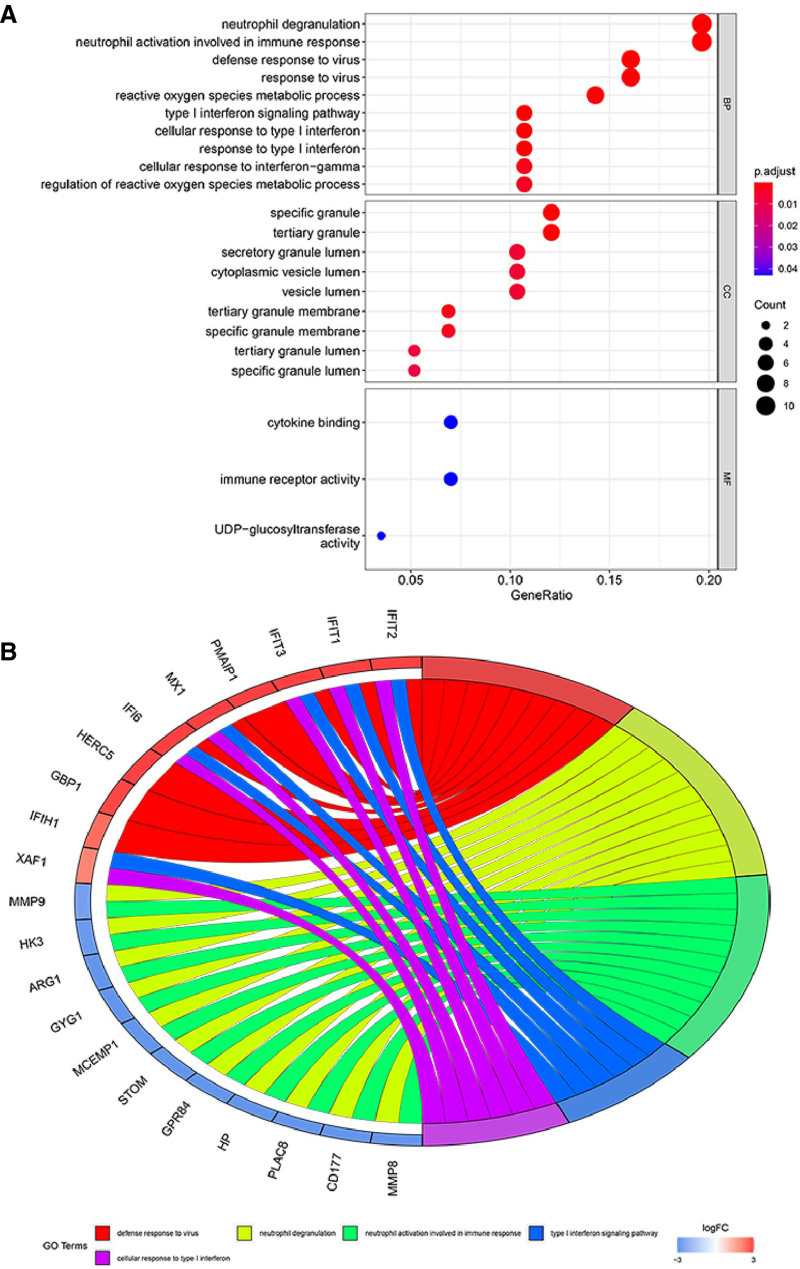
Visualization of GO term enrichment in DEGs. (A) Bubble chart representing the enriched Gene Ontology (GO) terms associated with the biological functions of differentially expressed genes (DEGs) in granulocytes from sepsis patients. The size of the bubble indicates the count of DEGs associated with each term, while the color gradient represents the adjusted *P*-value, demonstrating the significance of enrichment. The *x*-axis shows the gene ratio, indicating the proportion of DEGs associated with each GO term. Notably, terms related to neutrophil degranulation and immune system activation are prominently featured. (B) Circle chart displaying the relationship between DEGs and their associated GO terms across biological functions, cellular components, and molecular functions. Each segment of the circle corresponds to a specific GO term, with ribbons connecting the term to the respective DEGs. The width of the ribbon represents the gene count associated with each term, and the color coding corresponds to the different categories of GO terms, emphasizing the predominant association with neutrophil degranulation, specific and tertiary granules, cytokine binding, and immune receptor activity.

**Figure 6.**
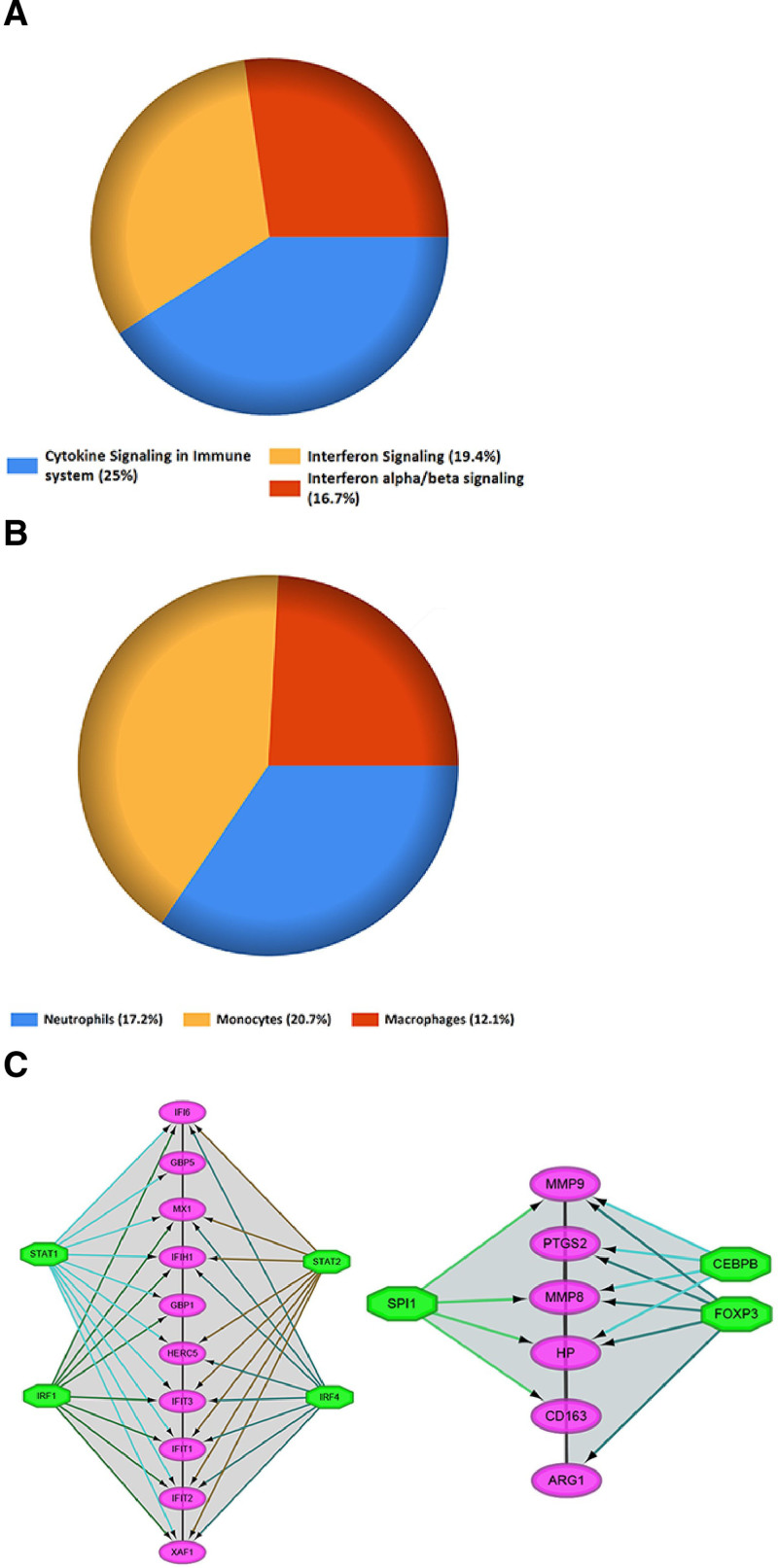
Pathway enrichment and transcription factor network analysis. (A) Pie chart illustrating the proportion of differentially expressed genes (DEGs) involved in key KEGG pathways. The chart highlights significant enrichment in cytokine signaling within the immune system (25%) and interferon signaling pathways (19.4%), reflecting the critical roles these pathways play in the immune response during sepsis. (B) Pie chart showing the cellular distribution of DEGs, with a significant presence in monocytes (20.7%) and neutrophils (17.2%). This distribution underscores the importance of these cell types in the pathophysiology of sepsis. (C) Network diagram presenting key transcription factors (TFs) that interact with the DEGs identified in the study. Nodes represent TFs and DEGs, with TFs including STAT1&2, IRF1&4, SPI1, CEBPB, and FOXP3 connected to the DEGs through lines indicating interactions. The network emphasizes the regulatory potential of these TFs in modulating gene expression during sepsis.

GO enrichment analysis indicated that the biological functions of the DEGs were predominantly related to neutrophil degranulation and immune system activation. In terms of cellular components, the DEGs were primarily associated with specific and tertiary granules, while molecular functions focused on cytokine binding and immune receptor activity (Fig. 5A and B). KEGG pathway analysis showed significant enrichment in cytokine signaling within the immune system (25%) and interferon signaling pathways (19.4%) (Fig. 6A). The DEGs were found to be primarily distributed among monocytes (20.7%) and neutrophils (17.2%) (Fig. 6B). Key transcription factors that were identified as interacting with these genes included STAT1&2, IRF1&4, SPI1, CEBPB, and FOXP3 (Fig. 6C).

## 4. Discussion

Sepsis remains a significant cause of mortality among hospitalized patients, further exacerbated by demographic shifts such as aging populations. Despite advancements in medical diagnostics and therapeutics, the prognosis for sepsis remains challenging, highlighting the importance of early detection and prompt treatment interventions, including fluid resuscitation and antibiotic therapy.^[1,10]^

The pathogenesis of sepsis involves a complex interplay of inflammatory and anti-inflammatory responses. Initially, the immune system mounts a hyper-inflammatory response to infection, which can shift towards immune paralysis if not resolved.^[8,11]^ Neutrophils play a vital role in this dynamic, acting as the primary cellular mediators in the innate immune response to pathogens. Optimal activation is crucial for effective pathogen clearance, but their overactivation can lead to tissue damage and systemic inflammation, predominantly through pathways mediated by toll-like receptors (TLRs) and the subsequent activation of NF-kB and MAPK pathways, resulting in the release of pro-inflammatory cytokines such as IL-1, IL-6, and TNF-α.^[12,13]^ This excessive inflammatory response can lead to a reduction in neutrophil TLR sensitivity and an increase in TLR inhibitors like NF-kBIA, further complicating the immune response.^[13]^

Furthermore, sepsis is characterized by an increase in nitric oxide production, which impairs neutrophil adhesion to endothelial cells, thus reducing their migration capacity and exacerbating vascular dysfunction.^[7]^ The release of immature neutrophils from the bone marrow during sepsis also contributes to disrupted microcirculation and organ dysfunction.^[14]^

This study’s bioinformatics analysis has identified significant alterations in the expression of genes such as MMP8, MMP9, and ARG1 in the granulocytes of patients with sepsis. These genes play a crucial role in regulating the immune response and neutrophil function. MMP8 and MMP9, in particular, are involved in the degradation of extracellular matrix components and are implicated in modulating inflammatory responses.^[15,16]^ Their involvement in septic responses, influenced by reactive oxygen species and other inflammatory mediators like TNF-α, highlights their potential as therapeutic targets.^[17]^

## 5. Conclusion

Our study underscores the significant potential of biomarkers such as MMP8, MMP9, and ARG1 in advancing the early diagnosis and targeted treatment of sepsis. These indicators not only enhance our understanding of the intricate pathophysiology of sepsis but also offer promising targets for therapeutic intervention. To establish their clinical utility, future research should focus on validating these biomarkers in larger and more diverse patient cohorts. Moreover, there is a need to explore how these biomarkers can be effectively integrated into routine clinical practices, with the potential to revolutionize the management and improving the outcomes of sepsis.^[10,15,17,18]^

## Acknowledgments

We extend our deepest gratitude to all the individuals who contributed to this study but did not meet the criteria for authorship. Special thanks are given to the technical staff and administrative support teams who played a critical role in the execution of this research. Their dedication and expertise were invaluable in facilitating the completion of this study. We would also like to express our heartfelt appreciation to all the participants of this study. Their willingness to contribute to scientific research during challenging times is deeply respected and appreciated. We sincerely wish all patients involved in this study a swift and complete recovery. Lastly, we acknowledge the support provided by the emergency ICU staff at Nantong Third People’s Hospital for their assistance in patient recruitment and sample collection.

## Author contributions

**Conceptualization:** Li Jin.

**Data curation:** Yuanyuan Wang, Mengxiao Jiang, Wenjie Liao.

**Formal analysis:** Li Jin, Feng Shao, Shengjie Zhang, Wenjie Liao.

**Investigation:** Li Jin, Wenjie Liao.

**Methodology:** Li Jin, Jun Qian.

**Validation:** Wenjie Liao.

**Writing – original draft:** Li Jin, Xiaowei He.
